# Neuropathology of 16p13.11 Deletion in Epilepsy

**DOI:** 10.1371/journal.pone.0034813

**Published:** 2012-04-16

**Authors:** Joan Y. W. Liu, Dalia Kasperavičiūtė, Lillian Martinian, Maria Thom, Sanjay M. Sisodiya

**Affiliations:** 1 Department of Clinical and Experimental Epilepsy, UCL Institute of Neurology and National Hospital for Neurology and Neurosurgery, Queen Square, London, United Kingdom; 2 Division of Neuropathology, National Hospital for Neurology and Neurosurgery, Queen Square, London, United Kingdom; 3 Epilepsy Society, Chalfont St Peter, Bucks, United Kingdom; Institut Jacques Monod, France

## Abstract

16p13.11 genomic copy number variants are implicated in several neuropsychiatric disorders, such as schizophrenia, autism, mental retardation, ADHD and epilepsy. The mechanisms leading to the diverse clinical manifestations of deletions and duplications at this locus are unknown. Most studies favour *NDE1* as the leading disease-causing candidate gene at 16p13.11. In epilepsy at least, the deletion does not appear to unmask recessive-acting mutations in *NDE1*, with haploinsufficiency and genetic modifiers being prime candidate disease mechanisms. *NDE1* encodes a protein critical to cell positioning during cortical development. As a first step, it is important to determine whether 16p13.11 copy number change translates to detectable brain structural alteration. We undertook detailed neuropathology on surgically resected brain tissue of two patients with intractable mesial temporal lobe epilepsy (MTLE), who had the same heterozygous *NDE1*-containing 800 kb 16p13.11 deletion, using routine histological stains and immunohistochemical markers against a range of layer-specific, white matter, neural precursor and migratory cell proteins, and NDE1 itself. Surgical temporal lobectomy samples from a MTLE case known not to have a deletion in *NDE1* and three non-epilepsy cases were included as disease controls. We found that apart from a 3 mm hamartia in the temporal cortex of one MTLE case with *NDE1* deletion and known hippocampal sclerosis in the other case, cortical lamination and cytoarchitecture were normal, with no differences between cases with deletion and disease controls. How 16p13.11 copy changes lead to a variety of brain diseases remains unclear, but at least in epilepsy, it would not seem to be through structural abnormality or dyslamination as judged by microscopy or immunohistochemistry. The need to integrate additional data with genetic findings to determine their significance will become more pressing as genetic technologies generate increasingly rich datasets. Detailed examination of brain tissue, where available, will be an important part of this process in neurogenetic disease specifically.

## Introduction

Copy number variants are insertions, deletions or duplications of segments of DNA. They can be found in healthy individuals, or be associated with disease. They have been the focus of considerable recent interest in neurological disorders [1], [2]. In particular, 16p13.11 copy number changes are associated with schizophrenia [3], mental retardation [4], [5], attention-deficit hyperactivity disorder [6] and both idiopathic generalised [7]–[9] and focal epilepsies [10]. In our previous study, we found 23/3812 patients had >100 kb deletions at 16p13.11, with Nuclear Distribution gene E homolog 1 (*NDE1*) involved in 22/23 [10]. *NDE1* encodes a protein that belongs to the highly-conserved nuclear distribution protein family. The Nde1 protein predominantly interacts both with Lissencephaly-1 (Lis-1), a protein essential for lamination of the cerebral cortex [11]–[13], and with dynein, a cytoplasmic motor protein that directs cellular cargos towards the ‘minus end’ of microtubules [11], [14]–[16]. Together, the Nde1-Lis-1-dynein complex mediates a range of intracellular motility activities, including the transport of organelles to the centrosomes, the orientation of mitotic spindles, the separation of chromosomes during mitosis and the assembly of centrosomes and spindles [13], [16]–[22], all of which are important for cortical development.

*NDE1* loss has been suggested a prime candidate for disease mechanisms associated with 16p13.11 deletion [4], [23]. Deficiency of Lis-1 and Nde1 expression in *Lis-1* and *Nde1* mutant mice and *Nde1* knockout mice results in a marked loss of cortical lamination, reduced brain size and a reduction in precursor cell division and segregation [11]–[13], [22]. In humans, *LIS1* haploinsufficiency (from a deletion at 17p13.3) results in Type 1 Lissencephaly or Miller Dieker syndrome, where patients have gross cortical dyslamination, a resulting smooth or under-folded cortex, and may experience epileptic seizures [24], [25]. Similarly, patients with homozygous frameshift mutation in *NDE1* show microlissencephaly (reduced brain size and simplified cortex) and experience seizures starting early in life [26], [27]. Heterozygous deletion of *NDE1* specifically may be a prime cause of epilepsy, both focal and idiopathic generalised, associated with 16p13.11 deletion [7], [10], [28]. Testing of this hypothesis directly in humans is difficult as the deletion is rare, and brain tissue is almost never available from patients with idiopathic generalised epilepsies. In our cohort of patients with focal epilepsy however, there were two patients with mesial temporal lobe epilepsy (MTLE) with *NDE1*-containing 16p13.11 deletion, who had previously undergone temporal lobectomy as treatment for their drug-resistant seizures. This surgically-resected material allows direct examination in the target tissue of the consequences of heterozygous *NDE1* deletion. We tested the hypothesis that *NDE1* loss would cause cortical dyslamination, using immunohistochemistry for a range of layer-specific, neural precursor and migratory cell markers.

## Results

The disease cases 1 and 2 both had the same 800 kb 16p13.11 deletion including *NDE1*.

### Histological staining

**Figure 1:**
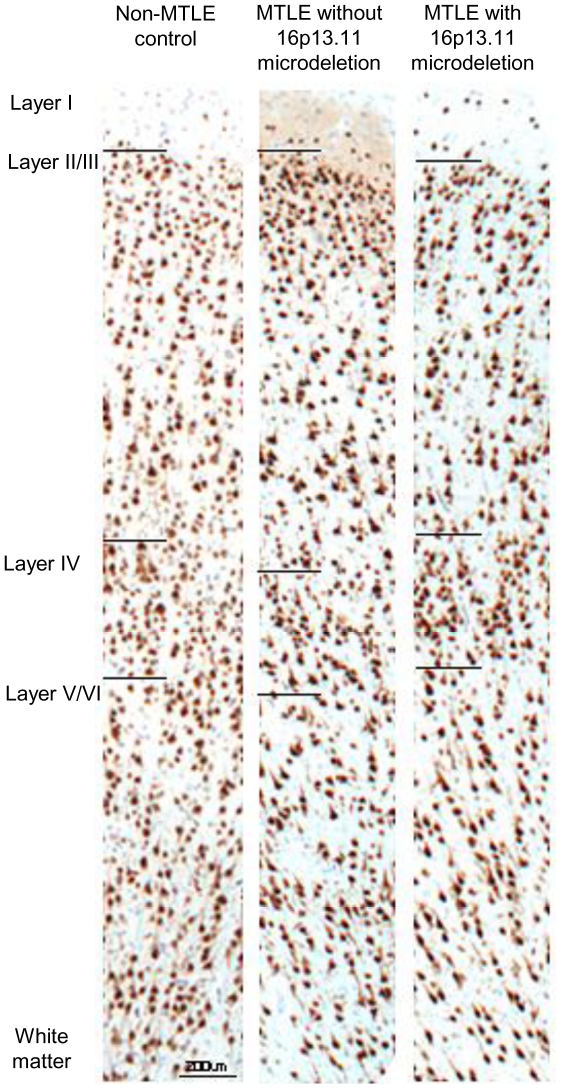
The temporal cortex of Case 2. The cortical lamination of the temporal cortex of Case 2 was normal, as compared to the MTLE case without *NDE1* deletion and non-epilepsy surgical controls. Scale = 200 µm.

**Figure 2:**
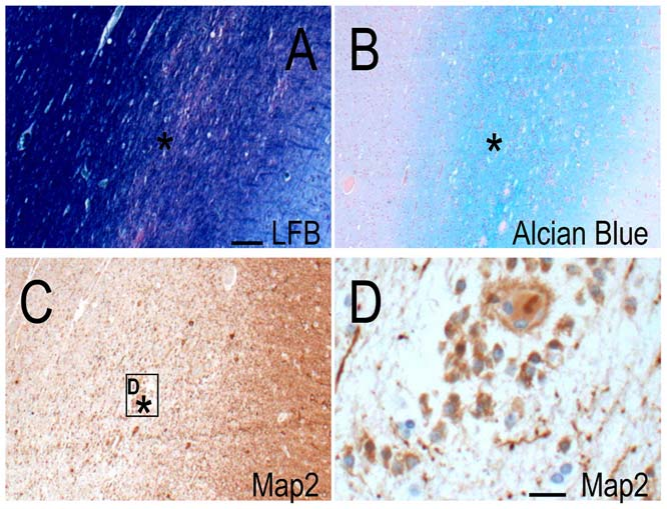
Hamartia in the temporal cortex of Case 1. LFB (A) and Alcian Blue (B) stains showed the location of the hamartia within the middle temporal gyrus (*). Small, round cells inside the hamartia were immunopositive for MAP2 as viewed at a low (C) and high (D) magnification. Scale = 100 µm (A–C), 20 µm (D).

The temporal cortex of Case 1, Case 2 and disease controls appeared normal with no cell loss or dyslamination evident in H+E, LFB or anti- Neuronal Nuclei (NeuN) labelled sections (Figure 1). LFB and Alcian Blue stains revealed a well-defined nodule of 3 mm in diameter, with an accumulation of metachromatic material, in the subcortical white matter of the middle temporal gyrus of Case 1 (Figure 2A–B). This nodule was composed of small, round cells that were immunopositive for microtubule-associated protein 2 (MAP2; Figure 2C–D) but not immunopositive for glial fibrillary acidic protein (GFAP), NeuN, synaptophysin or neurofilaments. These MAP2-immunopositive cells intermingled with larger, NeuN-immunopositive neurons and GFAP-immunopositive reactive astrocytes inside the nodule. Together, these neuropathological findings define the nodule as a hamartia (a small hamartoma [29], [30]). The overlying cortex appeared normal and serial sections taken from either side showed that the hamartia was neither part of a glio-neuronal tumour nor part of a larger malformation. Case 2 and other disease controls did not show pathological features indicative of a hamartia.

**Figure 3:**
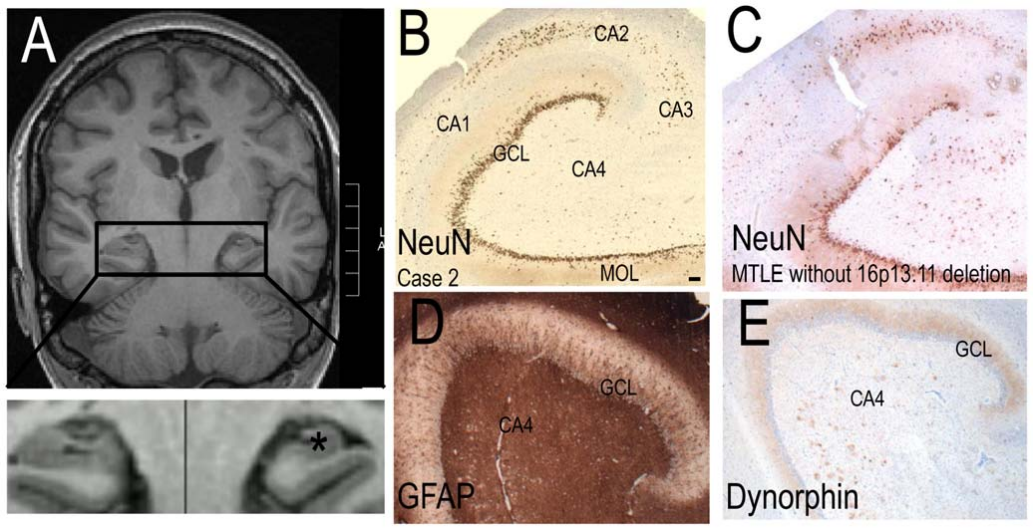
Hippocampal sclerosis in Case 2. (A) A coronal MRI image of Case 2 showing left hippocampal atrophy (*; inset). No cerebral malformations were evident. The histology showed hippocampal sclerosis: (B) NeuN-immunopositive cells were lost in the cornu Ammonis (CA) regions of the left hippocampus of Case 2, as seen in the MTLE control case without *NDE1* deletion (C). (D) Strong GFAP-immunoreactivity was observed in all regions of the hippocampus of Case 2. (E) Dynorphin-immunoreactivity was observed throughout the molecular layer of Case 2, demonstrating the presence of mossy fibre spouting. GCL = granule cell layer, MOL = molecular cell layer. Scale = 100 µm (B–E).

The hippocampus of Case 1 was normal, with no loss of cells in H+E, LFB and anti-NeuN labelled sections. Pre-operative magnetic resonance imaging (MRI) showed that Case 2 had a smaller left hippocampus compared to the right (Figure 3A). Microscopic examination of Case 2's surgically-resected left hippocampus revealed a marked loss of NeuN-immunopositive neurons in the cornu Ammonis (CA) 4, 3 and 1 (Figure 3B). The loss of NeuN-immunopositive cells in CA regions was also noted in the MTLE control without *NDE1* deletion (Figure 3C). Granule cell dispersion was present in Case 2. A dense matrix of GFAP-immunopositive cells and processes was observed throughout the hippocampus of Case 2, particularly in CA regions (Figure 3D). Dynorphin (Figure 3E) and neuropeptide Y immunoreactivities were evident in the inner, middle and outer molecular layers of the dentate gyrus of Case 2, confirming mossy fibre spouting. Together, these features were consistent with hippocampal sclerosis, a common pathology observed in MTLE [31].

### Layer-specific markers

**Figure 4:**
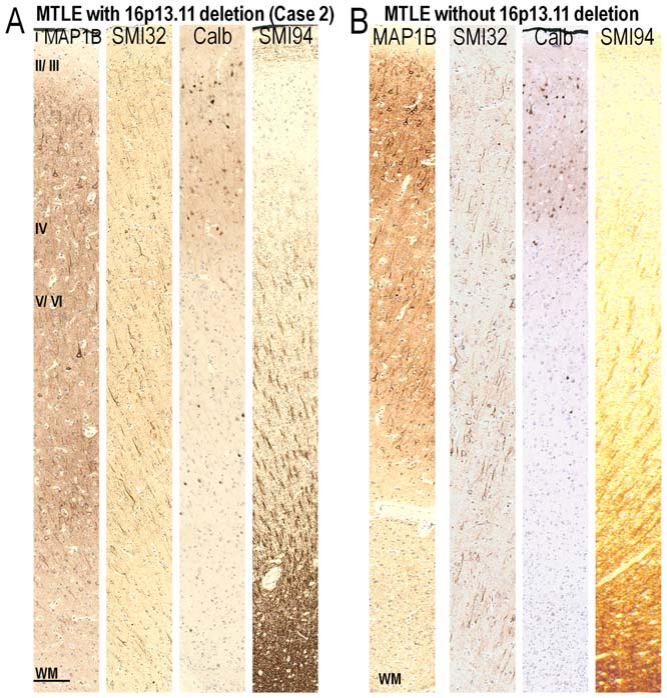
MAP1B, SMI32, Calbindin and SMI94-immunopositive labelling in the temporal cortex of Case 2 and the MTLE control. MAP1B and calbindin-immunopositive cells were predominantly observed in the upper cortical layers, while SMI32-immunopositive cells and processes were mainly found in the lower cortical layers of MTLE cases with (A) or without *NDE1* deletion (B). Immunoreactivity of SMI94 was observed in the lower cortical layers and white matter of both cases. Scale = 200 µm (A, B).

We examined the lamination of the temporal cortex and anatomy of the hippocampus of Case 1, Case 2 (Figure 4A, Figure S1A) and disease controls (Figure 4B, Figure S1B) using layer-specific immunohistochemical markers: anti-microtubule-associated protein 1B (MAP1B), N200, SMI32, anti-calretinin, anti-calbindin and anti-parvalbumin. These layer-specific markers label cell types specifically distributed within certain human cortical layers [32]–[35]. The white matter of Case 1, Case 2 and disease controls was investigated using SMI94 and anti-nogo-A antibodies. Nogo-A is a reticulin family protein expressed mostly by oligodendrocytes in the brain [36].

The pattern of immunolabelling with layer-specific and white matter markers was similar for Case 1, Case 2 and disease controls. In the temporal cortex, many MAP1B-, N200-, SMI32-, calretinin-, calbindin-, parvalbumin-immunopositive cells were observed in the upper cortical layers (II and III) of Case 1, Case 2 (Figure 4A, Figure S1A) and disease controls (Figure 4B, Figure S1B). A few MAP1B-, N200-, SMI32-, calbindin- and parvalbumin- immunopositive cells were also observed in the lower cortical layers (IV, V and/or VI) of all cases. All immunopositive cells in Case 1 and 2 appeared normal with no atypical or dysplastic features. Thick, SMI94-immunopositive radial fibres were predominantly observed from cortical layer IV to the white matter of all cases, with fewer horizontal fibres highlighted in layer I of Case 1, Case 2 (Figure 4A) and disease controls (Figure 4B). In the white matter of all cases, nogo-A-immunopositive cells were small and round, with labelling restricted to the scant cell body of oligodendrocytes. Nogo-A-immunopositive cells were found predominantly in the white matter rather than the gray matter.

**Figure 5:**
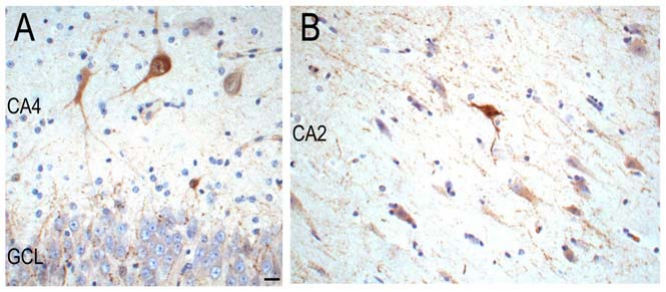
Parvalbumin-immunopositive labelling in the hippocampus of Case 2. A few numbers of parvalbumin-immunopositive cells were still observed in CA4 (A) and CA2 (B) of the sclerotic hippocampus of Case 2. Some parvalbumin-immunopositive processes and plexuses were observed around granule cells (A) and surviving pyramidal neurons in CA2 (B). CA = cornu Ammonis, GCL = granule cell layer. Scale = 20 µm (A, B).

In the hippocampus of all cases, MAP1B-, N200-, SMI32- calretinin-, calbindin- and parvalbumin-immunopositive cells were observed in the CA regions and subiculum. There were visibly fewer N200-, SMI32-, calretinin-, calbindin- and parvalbumin-immunopositive cells in the CA regions of Case 2 and the MTLE control without *NDE1* deletion, than in Case 1 and non-epilepsy controls, in keeping with the presence of hippocampal sclerosis in the MTLE disease control and Case 2. MAP1B- and calbindin-immunopositive granule cells were evident in the granule cell layer of all cases. Parvalbumin-immunopositive processes or synapses were often observed between granule cells in the GCL and the CA2 region of all cases (Figure 5). Nogo-A-immunopositive oligodendrocytes were evident in the white matter of all cases.

### Precursor and migratory cell markers

Antibodies against nestin, GFAPδand PAX6 label neural precursor cells [37]–[40], while antibodies against DCX and reelin label migratory cells in the developing and adult mammalian brain [41]–[43]. The immunolabelling of these markers was not markedly different between Case 1, Case 2 and disease controls.

**Figure 6:**
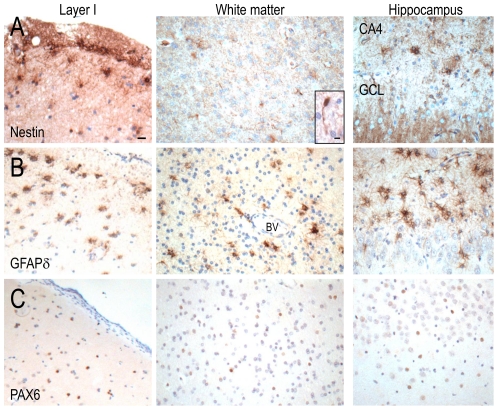
Nestin, GFAPδ and PAX6 immunopositive labelling in the temporal cortex and hippocampus of Case 2. Nestin- (A) and GFAPδ (B) strongly immunopositive glial-like cells were observed in cortical layer I and white matter of the temporal cortex and the hippocampus of Case 2. Nestin-immunopositive cells with short, unipolar processes were occasionally found in the white matter of Case 2 (A, middle inset). Small, round PAX6-immunopositive cells were found throughout the temporal cortex and hippocampus. BV = blood vessels, CA = cornu Ammonis, GCL = granule cell layer. Scale = 20 µm (A–C), 10 µm (A, middle inset).

**Figure 7:**
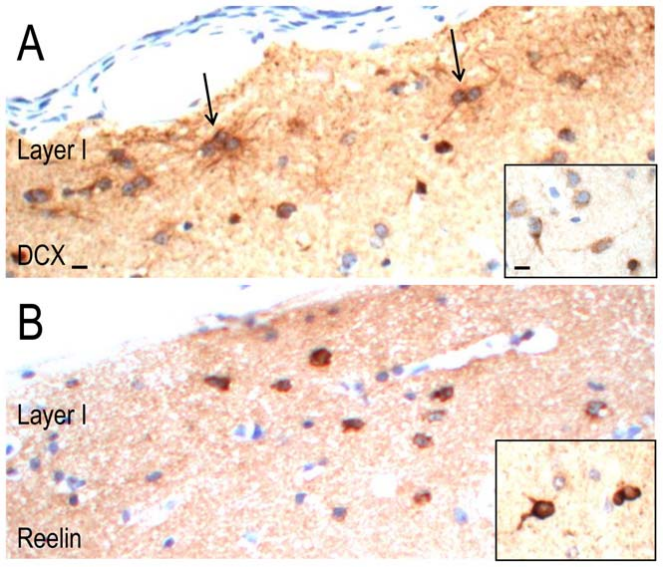
DCX and reelin-immunopositive labelling in the temporal cortex of Case 2. In cortical layer I, DCX-immunopositive cells with a glial-like morphology clustered in groups of two or three (arrows, A). DCX-immunopositive cells with short, bipolar processes were occasionally observed in the upper cortical layers (A inset). Reelin-immunopositive cells were also observed in cortical layer I (B) and in deeper cortical layers of Case 2 (B, inset). Scale = 10 µm (A, B, insets).

Nestin-, GFAPδ-, PAX6-, DCX- and reelin-immunopositive cells were predominantly observed in cortical layer I and white matter of Case 1, Case 2 (Figure 6A–C, 7A–B) and disease controls. GFAPδ- and DCX-immunopositive cells were binucleated or multinucleated with glial-like processes. Nestin-immunopositive blood vessels were observed throughout the cortex. A few GFAPδ-immunopositive cells were observed in the deeper cortex and in the white matter of all cases; they were often situated around blood vessels. PAX6- and small, reelin-immunopositive cells could also be seen in the other cortical layers of all cases.

In the hippocampus, GFAPδ-immunopositive cells were observed in all CA regions, particularly in the sclerotic hippocampus of Case 2 and the MTLE control case without *NDE1* deletion. PAX6- and DCX-immunopositive cells were observed throughout the hippocampus, particularly in the CA4 and granule cell layer of all cases. Large, reelin-immunopositive cells were observed in the CA4 region and in the molecular layer of the dentate gyrus, while small reelin-immunopositive cells were observed throughout the hippocampus.

### NDE1

**Figure 8:**
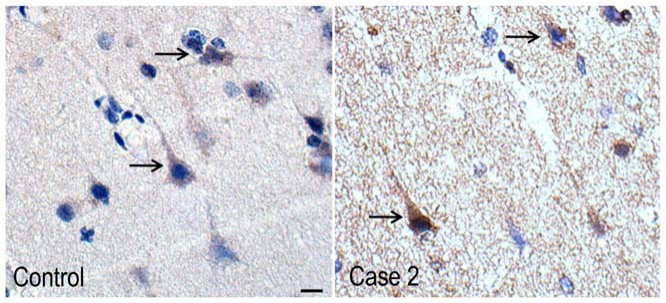
NDE1-immunopositive labelling in the temporal cortex of Case 2 and MTLE case without *NDE1* deletion. The immunoreactivity of NDE1 (arrows) was predominantly observed in the cytoplasm of neurons in Case 2 and the epilepsy control.

The immunoreactivity of NDE1 was observed in the cytoplasm of neurons throughout the temporal cortex and hippocampus of Case1 and 2 and disease controls (Figure 8). No marked difference in immunolabelling was observed between Case 1, Case 2 and disease controls.

### Multidrug resistance-related protein 1

**Figure 9:**
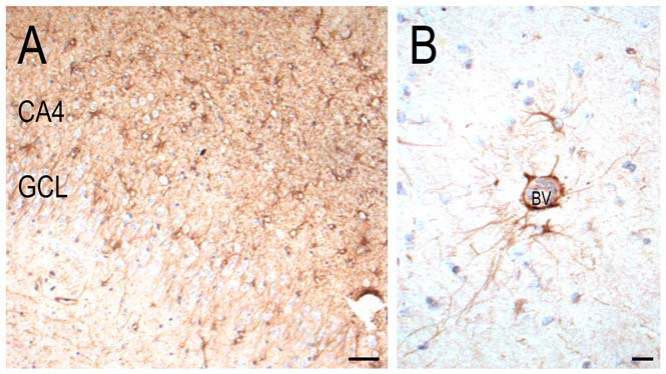
MRP1-immunopositive labelling in the temporal cortex and hippocampus of Case 2. (A) Many MRP1-immunopositive cells and processes were observed in the hippocampus. (B) MRP1-immunopositive cells were also evident in the temporal cortex (usually around blood vessels). CA = cornu Ammonis, GCL = granule cell layer, BV = blood vessel. Scale = 50 µm (A), 20 µm (B).

The chromosomal region 16p13.11 also contains *ABCC1*, which encodes the multidrug resistance-related protein 1 (MRP1). We investigated MRP1 expression using MRPr1, an antibody which has been used in previous human studies [44], [45]. MRPr1-immunopositive blood vessels were observed throughout temporal cortex and hippocampus of Case 1, Case 2 (Figure 9A–B) and disease controls. Many MRPr1- immunopositive glial cells and fibres were also observed in the cortical layer I and white matter of the temporal cortex and the hippocampal CA regions. Most MRPr1-immunopositive glial cells were found around blood vessels (Figure 9B). The glial expression of anti-MRP1 has been previously described in studies using surgical MTLE tissue without known *NDE1* deletion [44]. Disease controls showed a similar pattern of MRP1 immunoreactivity to that seen in Case 1 and Case 2.

## Discussion

Two key challenges in disease genetics are to identify pathogenic genetic changes and to understand how these changes lead to disease phenotypes. For neuropsychiatric diseases, the latter challenge is often especially daunting, as direct study of the organ involved, the brain, is usually impossible. But such studies, using brain material from patients with the neurological disease in question, especially those with known genetic abnormalities, are important as they may provide valuable insights and bounds about the effects of genetic variants on brain structure and disease mechanisms. We examined the surgically-resected temporal cortex and hippocampus of two people with MTLE with identical 800 kb deletion at 16p13.11, encompassing seven genes, including *NDE1* and *ABCC1*. In Case 1, the white matter of the temporal cortex contained a hamartia, which was not associated with any overlying cortical dyslamination or any dysmorphic neurons, balloon cells or more extensive changes seen in glio-neuronal tumours. In Case 2, we found neuronal cell loss, gliosis and re-organisation, changes typical of hippocampal sclerosis. The genetic basis, if any, of hippocampal sclerosis is unknown: in our previous study [10], only 2/23 patients with 16p13.11 deletions had hippocampal sclerosis, whilst other microdeletions are also associated with hippocampal sclerosis [46]. Both hamartia and hippocampal sclerosis occur in patients with drug-resistant epilepsy [30], [47], and are unlikely to be due to the 16p13.11 deletion.

Case 1 and Case 2 both had a normal, hexalaminar temporal cortex, housing appropriate layer-specific cell types. No marked difference was observed for NDE1 immunoreactivity or expression of proteins associated with cell division and migration in the temporal cortex or hippocampus of either case in comparison to disease controls. NDE1 immunoreactivity in both Case 1 and 2 and disease controls was localised to the cytoplasm of neurons, which is consistent with its role in intracellular transport [22], [48].

In animal studies, *Nde−/−* mutants have no *Nde1* expression and possess a brain that is a third smaller than controls, while heterozygous *Nde1+/−* mutant mice express a considerable amount of NDE1 protein, that is only slightly lower than controls in immunoblotting experiments, and these heterozygous *Nde1 +/−* mutant mice have brain size and weight comparable to controls [12]. Humans with 1 to 3.4 Mb homozygous deletions at 16p13.11 have small stature, dysmorphic features, seizures, microcephaly or microlissencephaly [4], [26]. In humans, the contrasting phenotypes of duplication and deletion suggest 16p13.11 is a dosage-sensitive region [28]. However, the resected brain tissue of our two patients with heterozygous 16p13.11 deletion was normal for the parameters we examined. Moreover, these patients did not have intellectual disability, or any remarkable abnormalities of appearance or stature. Therefore, no clear dose-dependency phenomenon is apparent in our two patients with, at least, loss of one copy of the whole *NDE1* gene. If 16p13.11 deletion contributes to brain disease in our two patients through loss of at least one copy of *NDE1*, as is the current favoured hypothesis, then this contribution to disease would not seem to be through pathology obvious at the light microscopic level or immunophenotypically-determined dyslamination. The deletion of *NDE1* could act at another, for example submicroscopic, level; it is also possible that the deletion unmasks a recessive mutation on the remaining allele, but this is unlikely given the normal appearance and cognitive abilities of our two patients and previous examination of this possibility [10]. Alternatively, loss of NDE1 in patients with heterozygous *NDE1* deletion may possibly be compensated by other proteins with similar functions such as LIS1 or NDE1-related protein 1 (NDEL1), a homolog of NDE1 [14], [15], [49].

Finally, it is possible that at least in our two patients, loss of NDE1 is not the key contributor to disease (here, epilepsy) associated with 16p13.11 deletion, suggesting that other candidates and mechanisms need to be sought. Overall, however, the link between heterozygous deletion of 16p13.11 and epilepsy, and other neuropsychiatric diseases, is clear [9], [10]: this deletion is not a polymorphism, and is at the very least a disease risk factor. In Case 1, seizures continued despite surgery, implying that epileptogenic tissue remained unresected. The 16p13.11 deletion in this case may therefore have conceivably acted as a risk factor for epilepsy by subtly altering brain structure beyond the resected tissue, a possibility we could not test as only resected tissue was available for analysis. We note, however, that *NDE1* is expressed in the human temporal lobe (e.g. [27], such that if *NDE1* deletion were acting by altering microscopic brain structure, examination for such changes in the temporal lobe would be a reasonable strategy: we did not find any alteration with the methods employed.

Our study illustrates the central role for detailed phenotyping in interpretation of genetic findings: for neurogenetics, human neuropathological studies are key, setting boundaries for putative mechanisms of action of identified genetic mutations. We anticipate an increasing role for such studies in human genetic diseases in general.

## Materials and Methods

**Figure 10:**
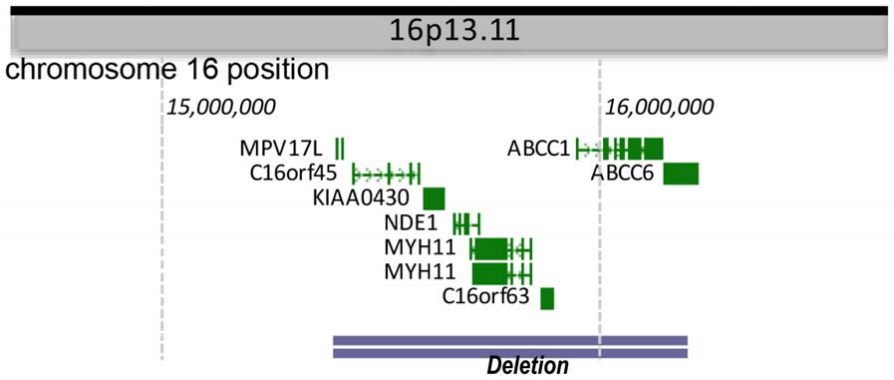
An illustration showing deleted genes in 16p13.11 in Case 1 and Case 2.

The study was approved by the Joint Research Ethics Committee of the Institute of Neurology and the National Hospital for Neurology and Neurosurgery. The work described has been carried out in accordance with The Code of Ethics of the World Medical Association (Declaration of Helsinki). Written informed consent was obtained for research. We examined surgically-resected temporal cortical and hippocampal tissue from two MTLE patients with confirmed 16p13.11 deletion. Their clinical details and results from genetic analysis are described in Table 1 and Figure 10. Neither of the patients has learning disability. No structural abnormalities were observed in MRI, apart from Case 2 who had a smaller left hippocampus compared to the right. Surgically-resected temporal cortex and hippocampus of a patient with MTLE known not to have the deletion of the entire *NDE1* gene, and resected neocortical tissue from three trauma patients were used as disease controls in immunohistochemical studies (Table 1). The three trauma patients were not tested for the 16p13.11 deletion, but the frequency of this deletion in patients with focal epilepsy was 0.6%, and the deletion was not observed in unselected controls [10], so it is very unlikely that all these three (or indeed any one) of the disease control patients had the deletion.

**Table 1:** Clinical details of cases.

MTLE cases with 16p13.11 deletion							
Identifier	Gender/Age	Deletion region (size)	Chromosome band	Genes	Surgery	Pathology	Surgery outcome
Case 1	M/36	chr16:15387380-16225138 (837,758)	16p13.11	*MPV17L, C16orf45, NDE1, MYH11, C16orf63, KIAA0430, ABCC1, ABCC6*	Hippocampal and temporal cortical resection	Hamartia in the subcortical white matter of the middle temporal gyrus. Not all hippocampal subfields were available for assessment.	Still experienced frequent seizures
Case 2	F/37	chr16:15387380-16225138 (837,758)	16p13.11	*MPV17L, C16orf45, NDE1, MYH11, C16orf63, KIAA0430, ABCC1, ABCC6*	Hippocampal and temporal cortical resection	Hippocampal sclerosis (cell loss and gliosis in CA1, 3 and 4)	Seizure free after surgery

Control cases			
MTLE case without 16p13.11 deletion			
Identifier	Gender	Age	Reason for surgery
N1	F	45	Epilepsy

Non-epilepsy cases			
N2	F	57	Head injury, brain swelling
N3	M	41	Head injury

All surgical tissue was fixed, processed and paraffin-embedded within one week following surgery. Routine histology staining, including haematoxylin and eosin (H+E) and luxol fast blue (LFB), was performed. Alcian Blue, an acid-mucosubstance stain, was used in Case 1 to investigate the observed hamartia. Automated immunohistochemistry was performed using Bond Max Automated Immunostainer and reagents (Leica, UK). 5 µm sections on adhesive microscopic slides (Raymond A Lamb, UK) were first processed in dewaxing solution and 100% alcohol. Antigen retrieval protocols were applied as detailed in Table 2 before sections were immersed in 3% hydrogen peroxide solution for 5 minutes. Sections were incubated with primary antibodies for 15 minutes. Post-primary polymer solutions were applied for 8 minutes. Chromogen activation was achieved using diaminobenzidine. Sections were dewaxed and rehydrated through xylene and graded alcohols and taken to water. Antigen retrieval procedures were applied as described in Table 2. All sections were outlined using a wax pen before placing flat in an immunohistochemical humidity chamber. Primary antibodies were diluted with Dako diluent and then incubated on sections as detailed in Table 2. NDE1 primary antibody was used at 1∶500. The following day, sections were incubated in Dako Envision horseradish peroxide solution for 30 minutes. Chromogen activation was performed using Dako Envision diaminobenzidine and substrate buffer for 5 minutes. Sections were washed in phosphate buffer solution between each step. Sections with omission of primary antibodies or their replacement non-specific IgG were included in experiments as negative control. Sections were then processed through graded alcohols and xylene and coverslipped (TissueTek). A light microscope and camera (Nikon Eclipse 80i and Sight DS Fi1) were used to visualize sections and to capture images. Montages were acquired using the JVC camera attached to an ImagePro Plus system (Media Cybernetics, USA). The specificity of the antibody against NDE1 was tested (see Figure S2).

**Table 2:** Primary antibodies used in the study.

Antibodies	Description	Source	Antibody: concentration and type	Mode of immuno-histochemical staining	Antigen retrieval protocol using Vector's unmasking buffer	Condition of Primary antibody incubation
N200	Layer specific cell marker	Sigma, USA	1∶3000 polyclonal	Manual	12 minutes, mw	1 hour, RT
SMI32	Layer specific cell marker	Covance, USA	1∶500 monoclonal	Manual	12 minutes, mw	1 hour, RT
Anti-MAP1B	Layer specific cell marker	Abcam, UK	1∶2500 monoclonal	Manual	15 minutes, mw	overnight, RT
Anti-calretinin	Layer specific cell marker	Sigma, USA	1∶3000 polyclonal	Manual	12 minutes,mw	overnight, RT
Anti-calbindin	Layer specific cell marker	Swant,Switzerland	1∶10000 polyclonal	Manual	12 minutes, mw	overnight, RT
Anti-parvalbumin	Layer specific cell marker	Swant , Switzerland	1∶5000 polyclonal	Manual	12 minutes, mw	overnight, RT
Anti-nestin	Neural precursor cell marker	Chemicon International, USA	1∶8000 polyclonal	Automated	20 minutes, mw	15 minutes, RT
Anti-PAX6	Neural precursor cell marker	Santa Cruz Biotechnology, USA	1∶50 monoclonal	Manual	15 minutes, mw	overnight, 4°C
Anti-GFAPδ	migratory cell marker	Chemicon International, USA	1∶5000 Polyclonal	Manual	12 minutes, mw	36 hours, 4°C
Anti-DCX	migratory cell marker	Abcam, UK	1∶4000 polyclonal	Manual	10 minutes, mw	overnight, RT
Anti-reelin	migratory cell marker	Chemicon International, USA	1∶1000 monoclonal	Manual	12 minutes	overnight, RT
SMI94	white matter marker	Covance, USA	1∶2000 monoclonal	Automated	10 minutes, enzyme treatment	15 minutes, RT
Anti-nogo-A	oligodendricytes marker	Chemicon International, USA	1∶500 polyclonal	Automated	20 minutes, enzyme treatment	15 minutes, RT
Anti-MRP1	ABC transporter marker	Alexis Biotechnology, USA	1∶30 monoclonal	Manual	6 minutes, EDTA buffer (Sigma, USA)	overnight, RT

mw = microwave; RT = room temperature.

## Supporting Information

Figure S1 **Immunoreactivities of layer-specific markers in the temporal cortex of Case 2 and the MTLE control.** MAP1B, calretinin, calbindin and parvalbumin-immunopositive cells were predominantly observed in the upper cortical layers, while N200- and SMI32-immunopositive cells and processes were mainly found in the lower cortical layers of MTLE cases with (A) or without *NDE1* deletion (B). Immunoreactivity of SMI94 was observed in the lower cortical layers and white matter of both cases. Scale = 200 µm (A, B).
(PDF)

Figure S2 **The specificity of the antibody against NDE1.** Immunoblotting experiment detected NDE1 at ∼38 kDa, the expected molecular weight of the protein, in the lysate from the resected temporal cortex of patient with mesial temporal lobe epilepsy.
(TIF)
