# Supplementary figures and images for: Neuropathology of 16p13.11 Deletion in Epilepsy
Source: PLoS One. 2012 Apr 16;7(4):e34813. doi: 10.1371/journal.pone.0034813 (PMC3327721; doi:10.1371/journal.pone.0034813)

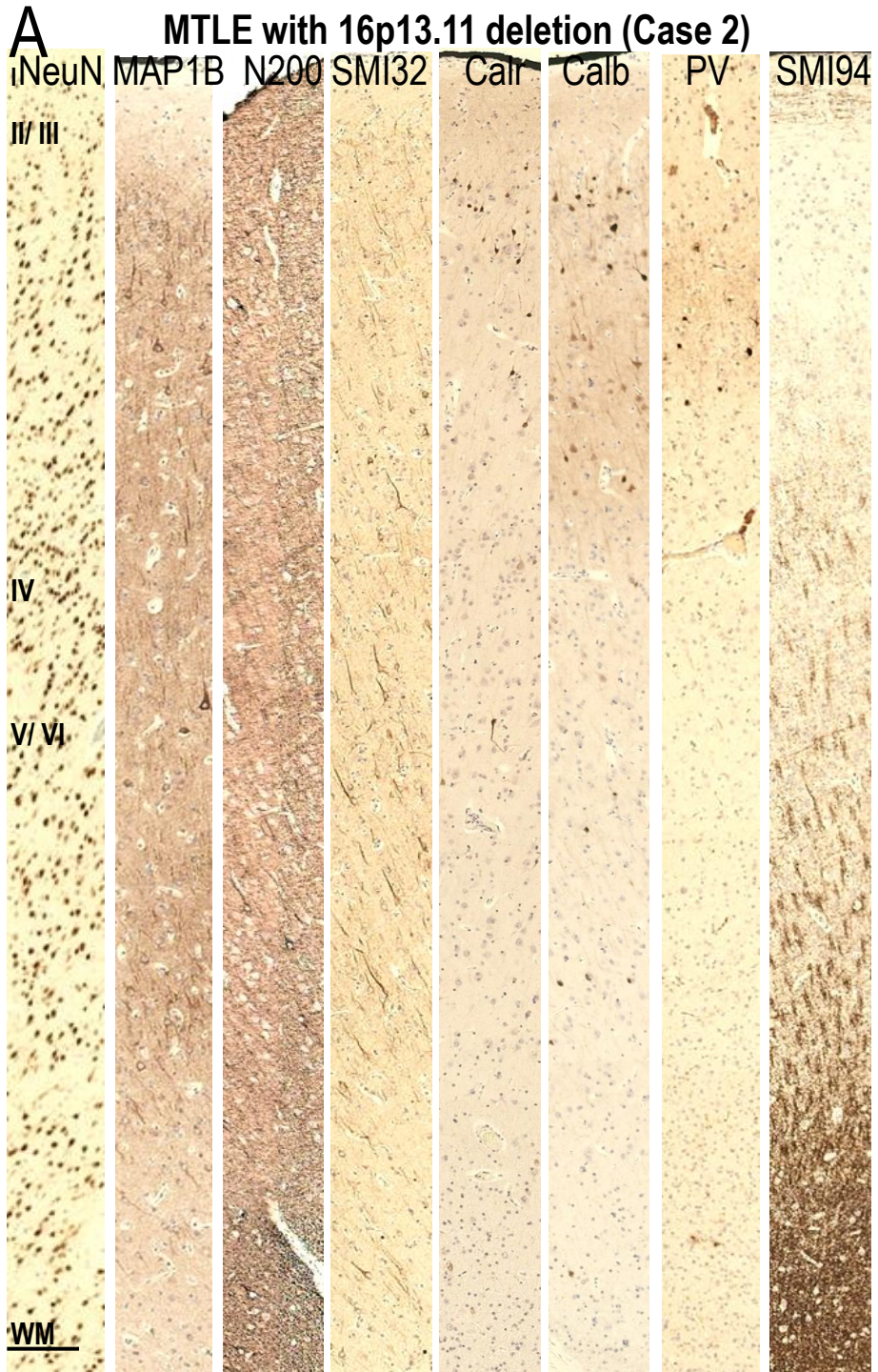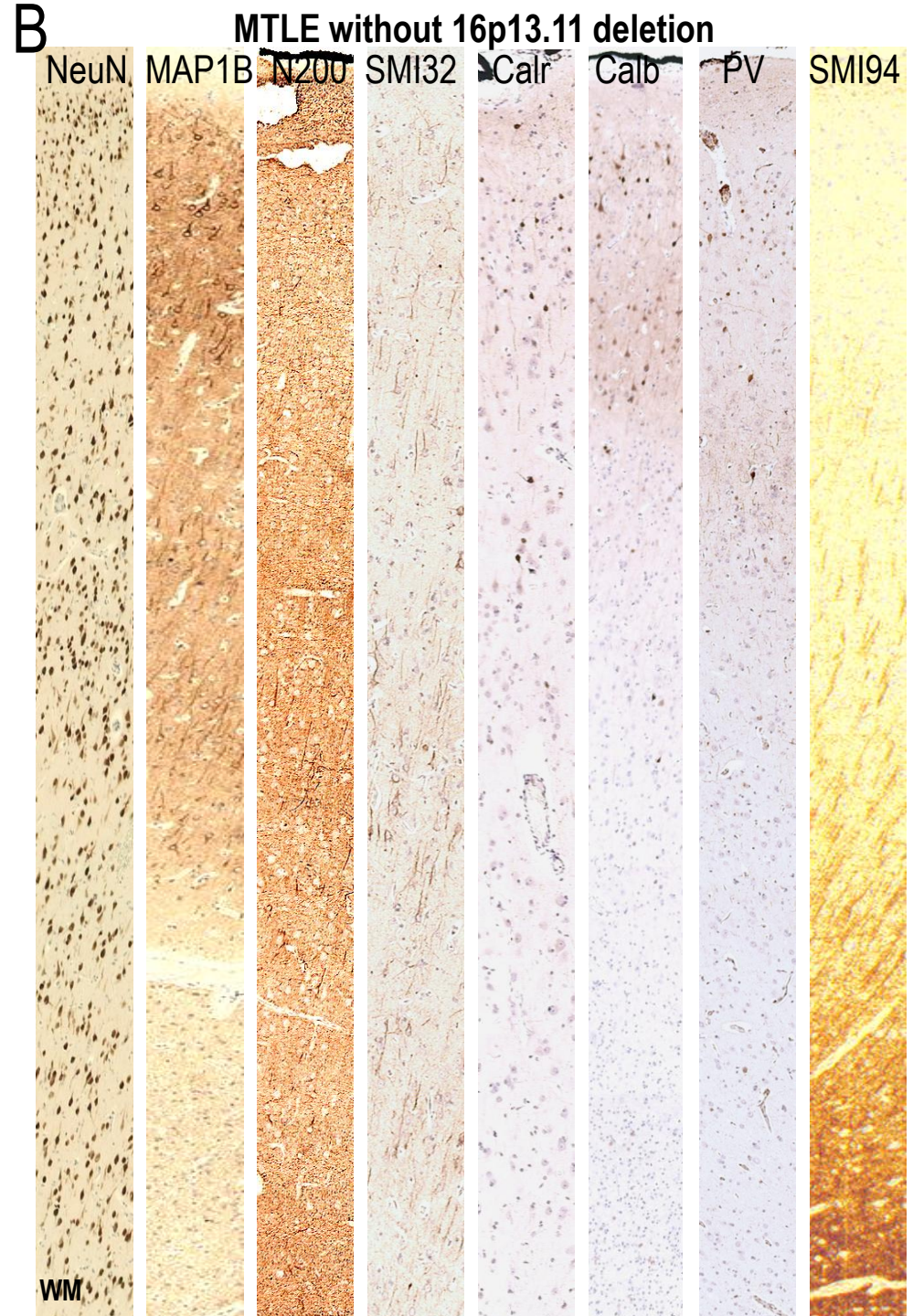

Supplement: Figure S1 — Immunoreactivities of layer-specific markers in the temporal cortex of Case 2 and the MTLE control. MAP1B, calretinin, calbindin and parvalbumin-immunopositive cells were predominantly observed in the upper cortical layers, while N200- and SMI32-immunopositive cells and processes were mainly found in the lower cortical layers of MTLE cases with (A) or without NDE1 deletion (B). Immunoreactivity of SMI94 was observed in the lower cortical layers and white matter of both cases. Scale = 200 µm (A, B). (PDF) [file pone.0034813.s001.pdf]

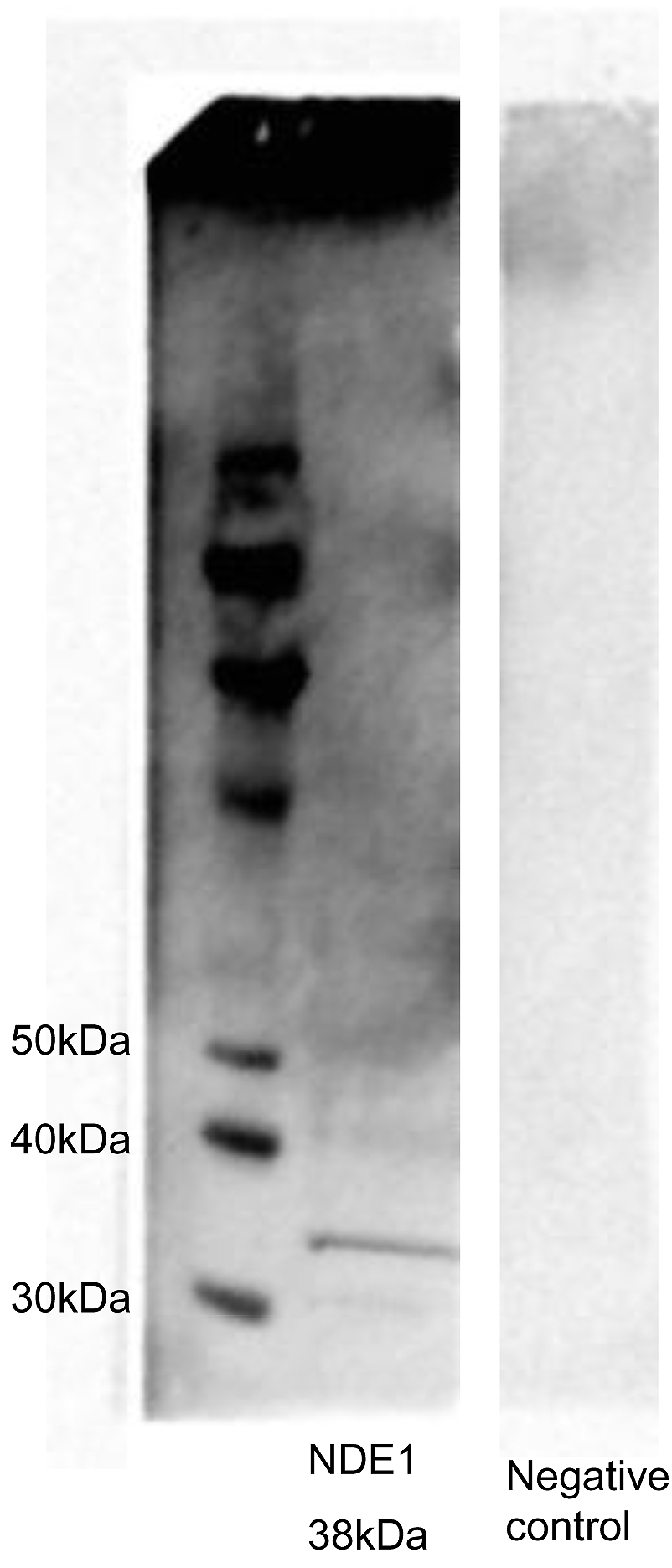

Supplement: Figure S2 — The specificity of the antibody against NDE1. Immunoblotting experiment detected NDE1 at ∼38 kDa, the expected molecular weight of the protein, in the lysate from the resected temporal cortex of patient with mesial temporal lobe epilepsy. (TIF) [file pone.0034813.s002.tif]
